# Special Issue: Diagnostic and Predictive Tissue Markers in G.I. Cancers

**DOI:** 10.3390/cancers15041329

**Published:** 2023-02-20

**Authors:** Stefano Chillotti, Francesco Vasuri

**Affiliations:** 1 Pathology Unit, IRCCS Azienda Ospedaliero-Universitaria di Bologna, 40138 Bologna, Italy; 2 School of Anatomic Pathology, Department of Biomedical and Neuromotor Sciences, University of Bologna, 40123 Bologna, Italy

The compelling advancements in systemic targeted therapies for cancer drastically changed the role of histopathological analyses in recent decades. Indeed, the identification and clinical application of new diagnostic and predictive markers for malignant neoplasms have become mandatory in order to offer the best standardization for patients’ prognosis and the best therapeutic options. The aim of this Special Issue was to focus on the identification, feasibility, and reproducibility of recent tissue markers in gastro-intestinal tumors.

These advancements in common knowledge can be achieved in two ways: by enhancing diagnostic methodologies and by discovering brand new markers for diagnosis and predictivity. 

As for methodology, the review from Renzulli et al. visibly reflects this need for new diagnostic and therapeutic tools from a radiologist’s point of view [1]: the authors extensively revised the available literature on the subject and found that while liver resection is the present-day standard of care in patients with cholangiocarcinoma (CCA), numerous locoregional approaches are available as an alternative therapy option in inoperable patients. In the present day, we have different techniques to choose from. The authors analyzed studies using interventional radiology approaches such as transarterial radio- and chemoembolization (TARE and TACE), photodynamic therapy (which remains the gold standard at the moment for unresectable CCAs), and microwave and radiofrequency ablation, but they also looked at other possibilities such as stereotactic external beam radiotherapy, proton beam treatment, internal radiotherapy, and endoscopic regional treatment. They found that all these therapeutic techniques can result in better regional control, particularly for combined hepatocellular–cholangiocellular carcinomas (cHCC-CCA) [2]. While these techniques are promising, there is a scarcity of clinical trials currently available, and as more trials will be published, there could be a notable impact on day-to-day practice. We want to comment on these propitious results by adding that, in our experience, we could see a probable improvement in the response rate of CCA patients when we are able to stratify these cancers more effectively in identifying which patients can benefit the most from these procedures. 

The work of Okuno et al. explores the diagnostic process in liver and bile duct malignancies from a completely different point of view [3]: the authors proposed an original methodology to increase the diagnostic sensitivity of cytology in biliary tree strictures, which currently represents the most common diagnostic tool, but it is beset by a low sensitivity for malignancy. The authors collected both the normal material used for cytology together with all the bile aspirated during the endoscopy. The bile was then left to sediment for 24 hours, and the liquid was decanted; the solids were then centrifuged, and a cell block was obtained. Using this novel method, the diagnostic material obtained was comparable in quantity and quality to an endoscopic biopsy, and even immunohistochemical studies were found to be reliable. All these benefits compounded helped the diagnostic sensitivity of the cell block method to jump to 62%. In our opinion this method is very promising and could especially help laboratories that are not able to perform routine FISH on cytology specimens. A possible concern could be how viable the material is for molecular analyses after the prolonged time from sampling to formalin fixation, so more studies are exhorted.

This Special Issue also focused on new and upcoming diagnostic and therapeutic biomarkers: In this regard, the review by Pekarek et al. introduced a very comprehensive state-of-the-art analysis on various up-and-coming biomarkers that are useful in the diagnosis and therapy of pancreatic carcinoma [4]. The authors presented some of the most promising blood biomarkers and compared them to the gold standard of serum Ca19-9 and in particular a combination of the dosing of this molecule: CEACAM1 was found to be potentially useful both in distinguishing healthy controls from patients with adenocarcinoma and in differentiating between chronic pancreatitis and adenocarcinoma [5]. Other blood markers were found to have limited use because of the relatively low specificity and sensitivity in the diagnostic workup of these patients. Pekarek et al. also presented the current situation regarding tissue markers, such as the methylation status of the tumour, which is correlated with exposition to exogenous agents and can alter the expression of several oncogenes and favour mutations in the KRAS pathway; unfortunately, it is of limited use in clinical practice and mostly remains as a useful research tool [6]. Some biomarkers that could prove useful in day-to-day practice in the near future are the ever-present mismatch repair genes, which can be found in up to 3% of the pancreatic cancers and can become a useful marker for immunotherapy, and the mutations of the BRCA1/2 genes that could make the tumour susceptible towards PPARP-inhibitor therapy [7]. The work by Pekarek et al. gives a good and ample picture of the landscape of biomarkers in pancreatic cancer that is proven to be a very fertile ground at the moment, with many possible new therapeutic predictors and diagnostic tools; however, the road to a well-established new marker is still in need of more research and confirmation studies.

The work by Salmikangas et al. brought us a new immunohistochemical (IHC) marker in the diagnosis of gastrointestinal stromal tumours (GIST): tensin-2 [8]. This protein is part of a family of cell adhesion molecules that is highly expressed in GIST. In daily practice, the most-used diagnostic IHCs for GIST are certainly c-KIT (CD117) and DOG-1, but these two markers come with limits. In particular, CD117 can be negative in up to 5% of GIST and DOG-1 can be positive in some sarcomas and even in gastric adenocarcinoma. The authors compared the IHC expression of tensin-2 in 175 GIST and 548 other sarcomas and found that strong staining for tensin-2 was present only in GIST, and 89.9% of the other sarcomas were completely negative for tensin-2; additionally, no GIST was found negative for this protein. The authors also tried to correlate the expression and staining intensity to the metastasis-free survival and overall survival, without finding a statistically significant difference.

Pezzuto et al. aimed to validate a ddPCR assay for the 124G>A mutation of a TERT promoter in hepatocellular carcinoma (HCC) in a local cohort of resected patients [9]. They compared their assay with standard Sanger sequencing and found a good correlation between the two methodologies. Interestingly, the authors also performed an analysis on the pathological and clinical characteristics of cancers and their mutational status. The results found that the TERT mutation was associated with a worse prognosis in terms of overall survival and that this specific mutation had a very high frequency, especially in virus-related (HCV) HCC, which represented most of the evaluated cases. We found these results very promising, because this mutation could be used as a prognostic marker in HCC, which represents a major cause of cancer-related mortality but it still suffers due to a lack of molecular markers [10]. Their cohort of patients consisted of mainly Child–Pugh A and B patients: we think that an enlargement of this cohort to also include more advanced patients, with different etiologies, and even a search of pre-operatory mutation status could very well implement the clinical practice.

Finally, Fernandez-Palanca et al. performed a review and meta-analysis of the literature regarding the presence of Neuropilin-1 (NRP1) overexpression in IHC as a pejorative marker in colorectal cancers (CRCs) and in primary liver cancers [11,12]: They collected and selected 15 studies on this argument and found that approximately half (50.40%) of the cases included in this meta-analysis exhibited NRP1 overexpression, with a significant association between high NRP1 levels and both enhanced tumor malignant characteristics and consistent shorter survival. NRP1 overexpression is a very promising marker in our opinion also because of its possibility to be used as a target for therapy [13]. We are of the opinion that a more thorough study of this marker in CCA could be especially beneficial for furthering therapy with respect to these patients, in which we are lacking, compared to other cancers, good theragnostic markers.

The papers included in this Special Issue depict a wide range of neoplastic processes that comprehended the gastrointestinal tract, liver, and pancreas and different methodologies ranging from imaging techniques to cytology and molecular biology. The portrait that emerged from these works is a promising effort towards a personalized approach for patients with gastrointestinal malignancies, encompassing all aspects of the diagnostic and predictive processes.
